# Loss of Survivin in the Prostate Epithelium Impedes Carcinogenesis in a Mouse Model of Prostate Adenocarcinoma

**DOI:** 10.1371/journal.pone.0069484

**Published:** 2013-07-31

**Authors:** Helty Adisetiyo, Mengmeng Liang, Chun-Peng Liao, Ari Aycock-Williams, Michael B. Cohen, Shili Xu, Nouri Neamati, Edward M. Conway, Chieh-Yang Cheng, Alexander Yu. Nikitin, Pradip Roy-Burman

**Affiliations:** 1 Genetic, Molecular and Cellular Biology Graduate Program, University of Southern California, Los Angeles, California, United States of America; 2 Department of Pathology, Keck School of Medicine, University of Southern California, Los Angeles, California, United States of America; 3 Department of Pathology, University of Utah, Salt Lake City, Utah, United States of America; 4 Department of Pharmacology and Pharmaceutical Sciences, School of Pharmacy, University of Southern California, Los Angeles, California, United States of America; 5 Centre for Blood Research, Department of Medicine, University of British Columbia, Vancouver, Canada; 6 Department of Biomedical Sciences, Cornell University, Ithaca, New York, United States of America; University of Colorado, United States of America

## Abstract

The inhibitor of apoptosis protein survivin is expressed in most cancers. Using the conditional *PTEN* deletion mouse model, we previously reported that survivin levels increase with prostate tumor growth. Here we evaluated the functional role of survivin in prostate tumor growth. First, we demonstrated that mice lacking the *survivin* gene in prostate epithelium were fertile and had normal prostate growth and development. We then serially, from about 10–56 weeks of age, evaluated histopathologic changes in the prostate of mice with *PTEN* deletion combined with *survivin* mono- or bi-allelic gene deletion. While within this time period most of the animals with wild-type or monoallelic survivin deletion developed adenocarcinomas, the most severe lesions in the biallelic survivin deleted mice were high-grade prostatic intra-epithelial neoplasia with distinct histopathology. Many atypical cells contained large hypertrophic cytoplasm and desmoplastic reaction in the prostatic intra-epithelial neoplasia lesions of this group was minimal until the late ages. A reduced proliferation index as well as apoptotic and senescent cells were detected in the lesions of mice with compound *PTEN/survivin* deficiency throughout the time points examined. *Survivin* deletion was also associated with reduced tumor expression of another inhibitor of apoptosis member, the X-linked inhibitor of apoptosis. Our findings suggest that survivin participates in the progression of prostatic intraepithelial neoplasia to adenocarcinoma, and that survivin interference at the prostatic intraepithelial neoplasia stages may be a potential therapeutic strategy to halt or delay further progression.

## Introduction

Survivin is a 142-amino acid residue protein that belongs to the family of inhibitor of apoptosis proteins (IAP). Due to its high expression in most human cancers and its role in promoting cell proliferation and inhibiting apoptosis, it is considered to be a potentially important therapeutic target [1]. It is thought that survivin over-expression might allow accumulation of mutations in transformed cells and thereby promoting tumor progression. Its expression is associated with increased resistance to cancer therapy-induced apoptosis and with lower patient survival [2]. Survivin contains a single baculoviral inhibitor of apoptosis repeat (BIR) domain and carboxyl terminal α-helix and takes form as a homodimer. Rather than binding directly to caspases, survivin blocks apoptosis by interacting with other partners including XIAP [3,4].

Transcription of the *Survivin* gene that is prominent in the mitosis phase of the cell cycle is also regulated by various growth factors and cytokines [5,6]. There is evidence that survivin also exists in the extracellular pool in the tumor microenvironment, and may be absorbed by cancer cells for their malignant progression [7]. Survivin’s differential subcellular localization is evidence of its multiple functions. Cytoplasmic/mitochondrial survivin is associated with a protective role against apoptosis, whereas nuclear survivin is proposed to be a regulator of cell division [8]. In normal cells its expression is at its highest in the G2/M phase of the cell cycle, but in tumors, it is reported to be independent of the cell cycle [9,10]. Survivin is a component of the chromosomal passenger complex (CPC), comprised of the Aurora B-kinase, Borealin, and INCENP. The CPC ensures proper attachment between the mitotic spindle and chromosomes and correct sister chromatid segregation, allowing successful cytokinesis [1]. In addition, survivin has been found to stabilize the mitotic spindle and mediate spindle assembly checkpoint [2].

Germline knockout of the survivin gene results in embryonic lethality [11], and its conditional knockout in thymocytes causes impaired cell proliferation, cell cycle arrest, mitotic spindle defects and apoptosis [12]; in neuronal precursors, it causes perinatal lethality and apoptosis [13]; in endothelial cells, it causes embryonic lethality [14]; and in hematopoietic progenitors, lack of survivin causes mortality due to bone marrow ablation, and erythropoiesis defects [15]. The role of survivin in the prostate gland, which primarily develops postnatally and which is a favored site for cancer in aging males, has not been previously investigated.

We have reported a strong expression of survivin protein in prostate cancer [16] of the conditional *Pten*-deletion mouse model [17–19] and in human prostate cancer specimens [20]. Interestingly, we also documented that certain extracellular signaling proteins, such as bone morphogenetic proteins BMP 2 and BMP7, continue increasing with the progression of prostate cancer in this mouse model, and that there is a direct relationship between BMP/Smad signaling and survivin up-regulation [16,21]. Additionally, we have identified Runx2, the master transcription factor for osteoblast differentiation as a key regulator of survivin transcription in prostate cancer cells, and observed that BMP signaling is also involved in up-regulation of Runx2 protein expression in these cells [16,20]. In this regard, it was interesting to note that in the conditional *Pten* deletion model of prostate cancer, protein levels of BMPs, Runx2, and survivin all increase with the tumor growth [16,20,21], implicating a potentially central role of survivin in prostate cancer. To determine the extent of survivin contribution to prostate tumor progression in this model system, we first document here that prostatic epithelium-specific deletion of *Survivin* has no significant effect on prostate organogenesis and function. Based on this finding, we proceeded to delete one or both alleles of *Survivin* in the *Pten* deletion model, and through analyses of these new strains we provide direct genetic evidence that loss of survivin expression in the prostate epithelium strongly inhibits the progression of prostatic premalignant lesions to adenocarcinoma in these animals.

## Materials and Methods

### Generation of prostate-specific Survivin*-*deleted or both Survivin- and Pten- deleted mice

For prostate epithelium-specific *Survivin* knockout we used *floxed Survivin* (*S*) allelic (*S* ^*f/f*^) female mice on a 129sv/Swiss background [22] and bred these with male *PB-Cre4* [23] transgenic mice (C57BL/6/DBA2), yielding progenies with heterozygous or homozygous deletion of *Survivin* (*cS* ^*+/-*^ *and cS* ^*-/-*^, respectively where *c* depicts Cre). Double deletion of *Pten* and *Survivin* in the prostate was generated by mating *S* ^*f/f*^ female mice with male mice carrying the *c* transgene and *Pten* ^*f/f*^ alleles on C57BL/6xDBA2/129 background [17]. All animals generated were of mixed genetic background. More detailed breeding schemes for various mouse genotypes are provided in the Supplementary Figure S1. Four distinct groups were generated: 1) Normal control group: contained the *floxed* alleles without *c*, abbreviated as *Pten* ^*f/f*^ *S* ^*f/f*^; 2) Control tumor group: *c; Pten* ^*f/f*^ *; S* ^*wild-type/wild-type*^ *or cPten* ^*-/-*^ *S* ^*+/+*^ ; 3) Experimental group with monoallelic deletion of *Survivin*: *c*; *Pten* ^*f/f*^ *; S* ^*wild-type/f*^ or *cPten* ^*-/-*^ *S* ^*+/-*^; and 4) Experimental group with biallelic deletion of *Survivin*: *c; Pten* ^*f/f*^ *; S* ^f/f^ *or cPten* ^*-/-*^ *S* ^*-/-*^. Animals were housed and maintained under identical conditions and animal experimentation was conducted in accordance to the ethical federal guidelines mandated by the University of Southern California Institutional Animal Care and Use Committee (Assurance Number: A-3518-01). Animal Protocols used for this study were approved by the University of Southern California Animal Care and Use Committee.

### Mouse genotyping

DNA was extracted from mouse tails and/or prostate tissues and subjected to PCR to determine the genotype*. Cre* was detected as a 500 bp fragment, while wild type and *floxed Survivin* was differentiated by the PCR products, 386 bp and 577 bp, respectively, detected using primers Adv 25 and Adv 28 [22]. Deletion of *Survivin* in the prostate was confirmed by the presence of 420-bp fragment generated with primers Adv17 and Adv28 as described [22]. Other primer sets were: *Cre* forward primer, GATCCTGGCAATTTCGGCTAT; and *Cre* reverse primer, TTGCCTGCATTACCGGTCGAT.

### Histopathology

Prostate tissues were collected from different age categories and incubated in 4% paraformaldehyde overnight at 4° C and then washed twice in PBS for 30 minutes before storing in 70% ethanol. Fixed tissues were processed by standard procedures, embedded in paraffin, cut to 5 µm sections onto glass microscope slides, and stained with hematoxylin and eosin after deparaffinization and rehydration [24,25]. The histopathology analysis was as thorough as possible as multiple sections of each prostatic lobe of these animals were examined microscopically.

### Immunohistochemical analysis

Paraffinized prostate tissue sections were deparaffinized and rehydrated before being subjected to antigen retrieval in 10 mM sodium citrate buffer at 95° C. Slides were then incubated in 1% H_2_O_2_ in methanol to block the endogenous peroxidase activity. Sections were blocked with 10% normal goat serum (Vector Laboratories) and 0.3% Triton X-100 in TBS for 1 hour at room temperature and incubated in primary antibody solutions overnight at 4° C. Antibodies used: Androgen receptor 1:200 (Santa Cruz Biotechnology), PTEN 1:400, Phospho-Akt 1:500, Cleaved Caspase-3 1:600, Phospho-H2AX Serine 139 1:1000 (Cell Signaling Technology), Ki67 1:400 (Vector Laboratories), p63 1:100 (Abcam), and Cytokeratin 8 1:50 (Development Studies Hybridoma Bank, IA, U.S.A.). Secondary antibodies used: affinity purified biotinylated rabbit anti rat IgG (H+L) and affinity purified biotinylated goat anti-rabbit IgG (H+L) (Vector Laboratories). Detection reagents: Vectastain Elite ABC kit (Vector Laboratories) and DAB (DAKO). The slides were counterstained with hematoxylin (Sigma), rinsed, and dehydrated before cover slips were placed over the tissue.

### Proliferation index

Tissue sections from four samples in each age group were stained with Ki67. Three random areas of each section were photographed at 400x magnification. Ki67 positive cells and total number of cells in each picture were counted using ImageJ software. Proliferation index was calculated as the number of Ki67 positive cells divided by the total number of cells.

### Cleaved Caspase-3 quantitation

Tissue sections from at least three samples from each genotype: *cPten* ^*-/-*^ *S* ^*+/+*^, *cPten* ^*-/-*^ *S* ^*+/-*^, and *cPten* ^*-/-*^ *S* ^*-/-*^ ranked with different degrees of PINs were stained with cleaved Caspase-3 antibody. Three random areas of each section were photographed at 400x magnification. Cleaved Caspase-3 positive cells and total numbers of cells in each picture were counted using ImageJ software. Extent of apoptosis was calculated as the number of cleaved Caspase-3 positive cells divided by the total number of cells.

### Assessment of γ-H2AX staining

Prostate tissue sections from mice at different age groups (10, 21, and 37 weeks) and of various genotypes (*cPten* ^*-/-*^ *S* ^*+/+*^, *cPten* ^*-/-*^ *S* ^*+/-*^, and *cPten* ^*-/-*^ *S* ^*-/-*^) were stained with phosphorylated H2AX (γ-H2AX) antibody. Three random areas of each section were photographed at 400x magnification. γ-H2AX positive cells and total numbers of cells in each picture were counted using ImageJ software. Mean ratio of cells with DNA fragmentation was calculated as the number of γ-H2AX positive cells divided by the total number of cells.

### Senescence-associated β-Galactosidase staining

Prostate tissue samples were embedded in OCT, frozen on dry ice, sectioned to 8 µm, set on microscope glass slides and air-dried. The sections were then fixed in 0.5% glutaraldehyde in PBS for 15 minutes at room temperature and washed twice in PBS at room temperature. The slides were stained in β-Galactosidase staining solution (X-Gal, NaCl, MgCl_2_, Fe II, and Fe III in phosphate citrate buffer pH 6.0) for 8 hours in a 37° C incubator [26]. The samples were then washed three times in PBS until no longer yellow and counter-stained with 0.1% Nuclear Fast Red (Sigma) in 5% aluminum sulfate, rinsed, and dehydrated before mounting medium was added to the slides to hold the cover slips.

### Western blot analysis

Prostate tissues were pulverized and then lysed using RIPA buffer (Sigma). Protein concentration was determined by the BCA protein assay method (Pierce). 5 µg of protein was loaded in each lane and subjected to Western blot analysis. Antibodies used: Survivin, XIAP, and Livin 1:2000 (Cell Signaling Technology). β-Actin 1:1000 (Santa Cruz Biotechnology, Inc.) was used as loading control.

### Statistical analysis

Statistical comparisons were established using an unpaired, two-tailed *t* test. A minimum of three independent analyses were carried out for each experiment. Statistical significance is determined by *p*-value < 0.05.

## Results

### Murine prostate organogenesis and growth are not impaired by the loss of survivin in the prostatic epithelium

**Figure 1:**
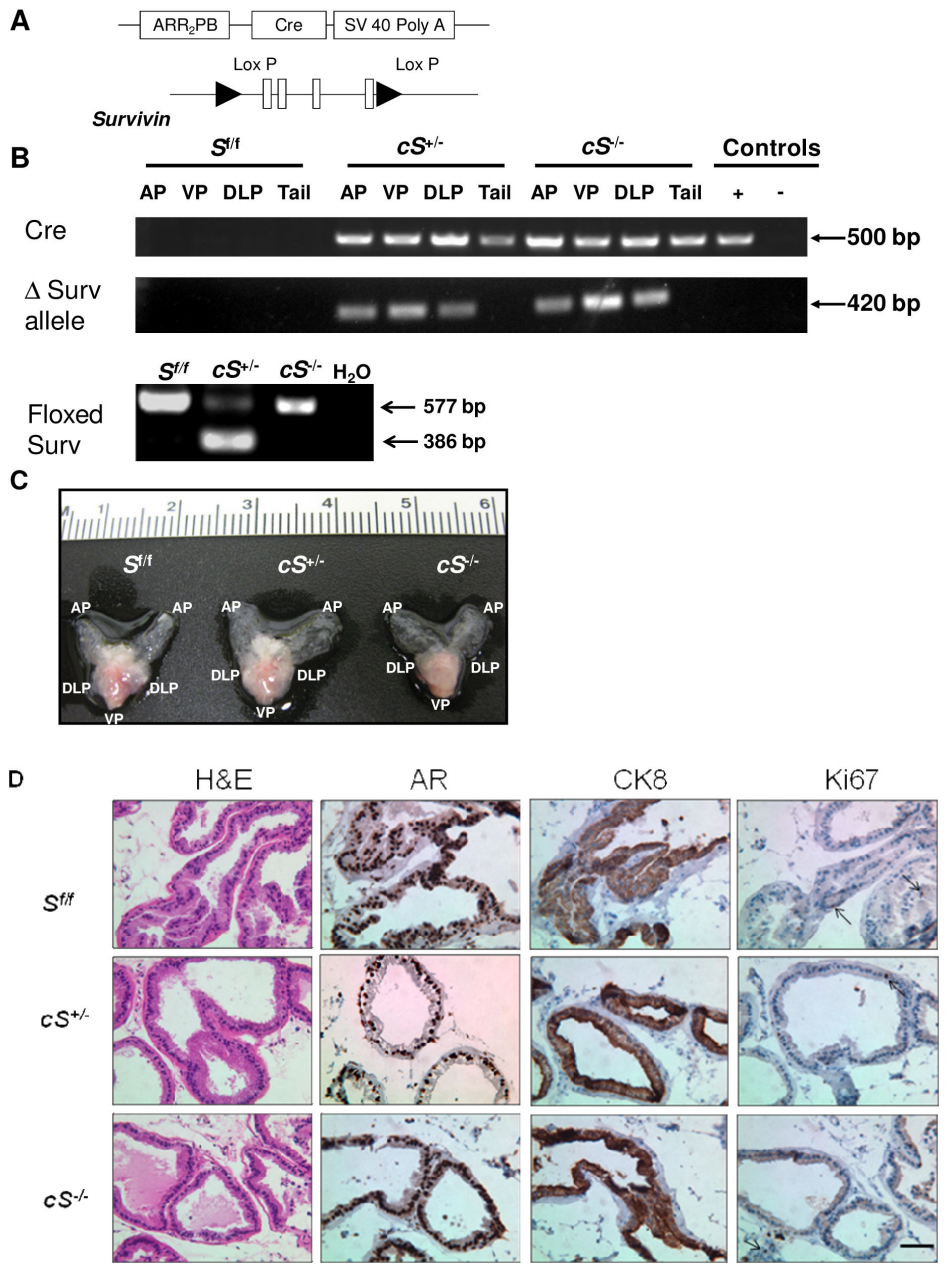
*Survivin* deletion has no effect on normal prostate development. (A) Prostate epithelium-specific *Survivin* deletion was produced by homologous recombination via ARR2PB promoter-driven Cre expression and Lox P sites flanking all four exons of *Survivin*. (B) Illustration of PCR analysis for ascertaining genotypes, in this case, using tissues from 20-week old mice. Tail DNA extracts from cPten^-/-^ and *Pten* ^*f/f*^ mice were used as positive (+) and negative (-) controls for Cre. H_2_O, water; AP, anterior prostate; VP, ventral prostate; DLP, dorsolateral prostate. (C) Ventral view of prostate showing generally normal gross morphology whether with either single or biallelic inactivation of *Survivin*. (D) Examples of H&E or immunostaining for androgen receptor (AR), luminal epithelial cell marker cytokeratin 8 (CK8), and proliferation marker Ki67, using ventral prostate lobes collected from 20-week old mice. Positive expression is indicated by the brown staining in the cytoplasm (CK8) or nucleus (AR and Ki67). The results illustrate retention of normal tissue morphology, protein marker expression pattern, and proliferation rate in prostates with heterozygous or homozygous deletion of *Survivin*. Bar, 50 µm.

*Survivin* gene deletion in the prostate of male mice was accomplished using the *Cre-Lox P* system. Male mice expressing the *PB*-*Cre4* transgene [23] driven by the prostate-epithelium-specific rat *ARR*_*2*_ *PB* promoter [27] were crossed with female *Survivinfloxed* (*S* ^*f/f*^) mice to yield progeny with *S* ^*f/f*^ *, cS* ^*+/-*^, and *cS* ^*-/-*^ genotypes (Figure 1A, Figure S1A). The genotype of each mouse was determined by PCR as described in Methods. Deletion of survivin was confirmed by the presence of a 420 bp band on the gel (Figure 1B). Murine prostates were collected at various time points of about 8, 20, 36, and 52 weeks, and there was no significant difference observed in the gross morphology of the prostates between *S* ^*f/f*^ *, cS* ^*+/-*^, and *cS* ^*-/-*^ mice in all age groups. All prostates appeared normal and were similar in size (Figure 1C). Furthermore, histopathological and immunohistochemical analyses did not detect any abnormalities in the morphology and marker protein expression pattern of prostatic glandular structures of mice with single and double *Survivin* allelic deletion (Figure 1D). The proliferation marker Ki67 expression level also did not deviate from normal upon *Survivin* deletion (Figure 1D). Thus, *Survivin* deletion appeared not to affect normal prostate organogenesis and growth. A number of mature male *cS* ^*-/-*^ were used for breeding and were determined to be fertile, with the size of litters produced falling within the normal range.

### Loss of survivin in prostate of the conditional Pten-deletion mouse model inhibits tumor progression

**Figure 2:**
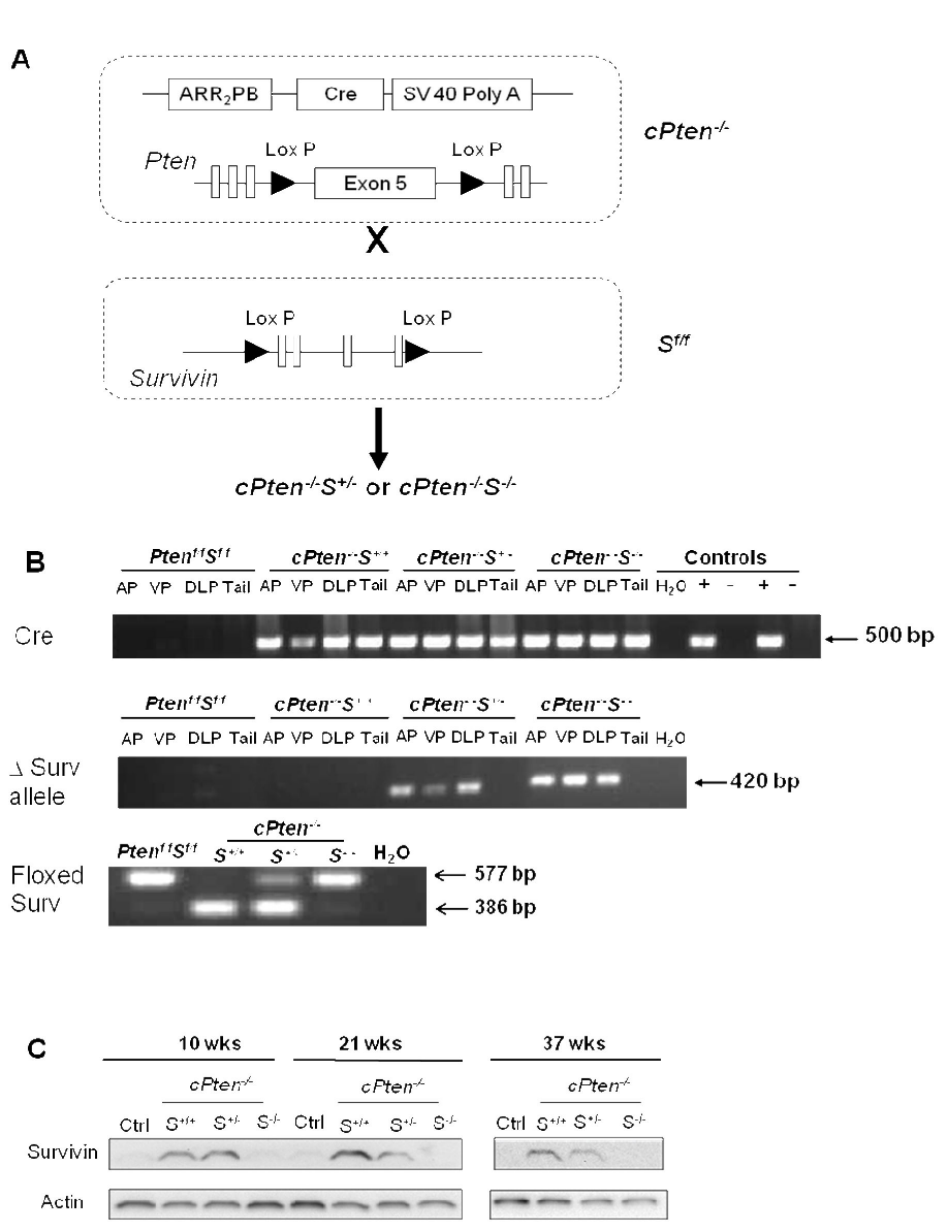
Generation of double conditional knockout mice lacking alleles of *Pten* and *Survivin*. (A) Mouse genotype of interest was obtained by crossing mice carrying Cre transgene and floxed phosphatase region of *Pten* (exon V) with *floxed Survivin* mice. (B) Genotypes of mice (shown: 10 weeks old) were determined by PCR analysis of the tissue samples. Tail DNA extracts from cPten^-/-^ and *Pten* ^*f/f*^ mice were used as positive (+) and negative (-) controls for Cre, H_2_O, water. (C) Status of *Survivin* deletion was also confirmed at the protein level. This is illustrated by a representative Western blot from the ventral prostate of mice from the 10-, 21-, and 37-week groups, with β-actin serving as loading control. Prostate tissue lysates from *Pten* ^*f/f*^ *S* ^f/f^ mice were used as normal control (ctrl).

For this study we established a prostate-specific *Pten* and *Survivin* double knockout mouse strain as illustrated in Figure 2A. The complete breeding schematic is outlined in Figure S1B. Mice with the following genotypes, confirmed with PCR analysis, were included in our study: *Pten* ^*f/f*^ *S* ^f/f^, *cPten* ^*-/-*^ *S* ^*+/+*^, *cPten* ^*-/-*^ *S* ^*+/-*^, and *cPten* ^*-/-*^ *S* ^*-/-*^. The presence of *Cre* was ascertained by a 500 bp fragment, and by a 386 bp product for wild type *Survivin* and a 577 bp fragment representing *floxed Survivin* (Figure 2B). Deletion of *Survivin* alleles was assessed by the presence of a 420 bp band on the gel as well as significant lack of detection of the corresponding protein on the Western blots (Figure 2B).

**Figure 3:**
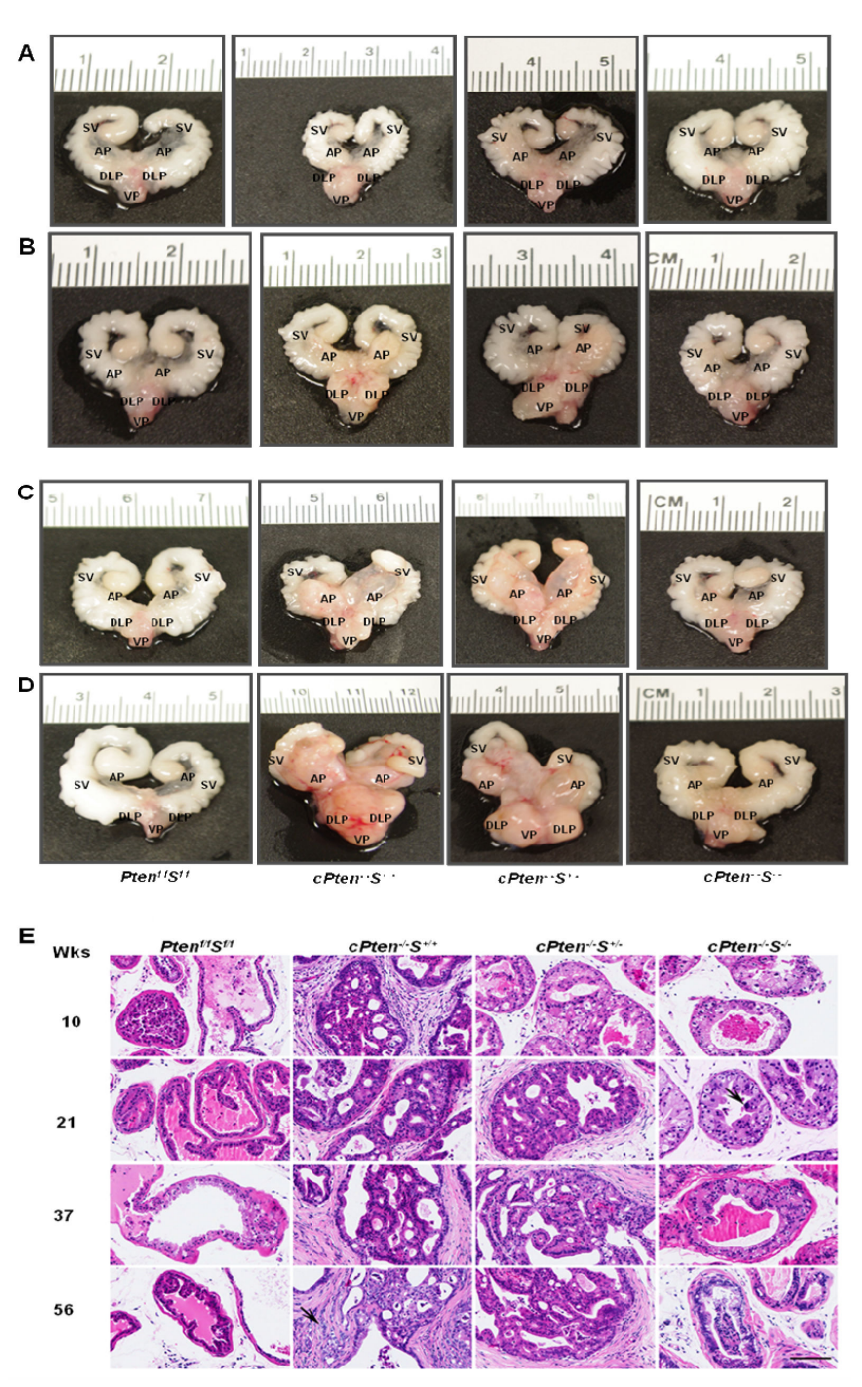
Loss of *Survivin* in conditional *Pten* deletion mouse model delays prostate tumor progression. Representative ventral view of prostate of various genotypes at different time points of aging, (A), (B), (C), (D) indicating animals from the 10-, 21-, 37-, 56-week groups, respectively. Deletion of both *Survivin* alleles yielded a significantly smaller prostate relative to the sizes seen in the other groups. AP, VP, DLP as in Figure 1; SV, seminal vesicles. (E) Representative depiction of histological analysis of H&E staining of paraffin-embedded prostate tissue sections of mice from 10, 21, 37, and 56 week-old groups: *cPten* ^*-/-*^ *S* ^*+/+*^ mice appeared to develop high grade PINs [28–30] in the majority of glands as early as 9 weeks of age. At later time points invasive adenocarcinomas (arrow, 56 weeks) could be detected. *cPten* ^*-/-*^ *S* ^*+/-*^ mice of the 10-week group displayed PINs 1-2, followed by detection of PINs 3-4 in the 21-week age group. Prostate epithelium of *cPten* ^*-/-*^ *S* ^*-/-*^ mice encompasses single atypical cells containing large hyperchromatic nuclei (arrow, 20 weeks) and large cytoplasm between 8 and 20 weeks of age. PINs 2-3 were observed in some of the glands from the 37- and 56-week age groups. Note that desmoplastic reaction was absent or minimal in lesions of *cPten* ^*-/-*^ *S* ^*-/-*^ mice. Dorsolateral lobes. Bar, 50 µm.

The gross morphology of the prostate glands of the *Pten* ^*f/f*^ *S* ^*f/f*^ *, cPten* ^*-/-*^ *S* ^*+/+*^ *, cPten* ^*-/-*^ *S* ^*+/-*^, and *cPten* ^*-/-*^ *S* ^*-/-*^ mice was examined. Starting at 10 weeks (range 9-11 weeks), prostates collected from *cPten* ^*-/-*^S^+*/*+^ mice already exhibited abnormality by their whitish, denser appearance compared to the translucent, smaller normal prostate. While *cPten* ^*-/-*^ *S* ^*+/-*^ seemed to exhibit a gross morphology similar to the *cPten* ^*-/-*^ *S* ^*+/+*^ animals, the gland of the *cPten* ^*-/-*^S^-*/*-^ mice displayed a normal morphology (Figure 3A). Although by 21 weeks (range 19-23 weeks) of age *cPten* ^*-/-*^S^-*/*-^ prostates no longer looked normal, still the gross appearance reflected a smaller size and less denser glands relative to those of the *cPten* ^*-/-*^ *S* ^*+/+*^ *and cPten* ^*-/-*^ *S* ^*+/-*^ animals at the corresponding age (Figure 3B). This pattern continued through 37 weeks (range 34-41 weeks) to the last time point of observation, 56 weeks (range 51-62 weeks), with *cPten* ^*-/-*^S^+*/*+^ and *cPten* ^*-/-*^ *S* ^*+/-*^ displaying a similar extent of enlargement of the prostate, much more so than that of *cPten* ^*-/-*^S^-*/*-^ (Figure 3C, 3D).

Histological and immunohistochemical analyses indicated the nature of the lesions formed in the presence or absence of survivin expression in the conditional *Pten* deletion model. By 10 weeks, 3 of 4 of the *cPten* ^*-/-*^S^+*/*+^ mice displayed high grade prostatic intraepithelial neoplasia (PIN), which could be identified as PIN3 or 4, while only one out of 4 of the *cPten* ^*-/-*^ *S* ^*+/-*^ and none of *cPten* ^*-/-*^S^-*/*-^ mice exhibited this phenotype at this time point (Figure 3E, Table 1). At 21 weeks, however, high grade PIN lesions were observed in all prostate glands of *cPten* ^*-/-*^ *S* ^*+/-*^ (Figure 3E). Single atypical cells with large heterochromatic nuclei and large cytoplasm were found in prostate specimens of *cPten* ^*-/-*^S^-*/*-^ mice between 10 and 21 weeks of age, and PIN 2 (low grade PIN) and PIN 3 were detected in some glands after further aging (Figure 3E, Table 2). As early as 10 weeks, one out of 4 *cPten* ^*-/-*^ *S* ^*+/+*^ mice developed early carcinoma characterized by microinvasion, while none such lesions were observed in the *cPten* ^*-/-*^ *S* ^*+/-*^ or *cPten* ^*-/-*^S^-*/*-^ group at this age. Appearance of early carcinoma lesions was detected in the single *Survivin* allelic deletion in the *Pten* null prostate at 21-week time point. The incidence and severity of carcinoma (early to adenocarcinoma) progressively increased up to 56 weeks in both the *cPten* ^*-/-*^ *S* ^*+/+*^ and *cPten* ^*-/-*^S^+*/*-^ mice (Table 1, Tables S1 and S2). In contrast, no indications of early carcinoma or adenocarcinoma were found in the prostates of the *cPten* ^*-/-*^S^-*/*-^ mice at any of the time points analyzed. The most severe diagnosis that could be assigned to these mice at 56 weeks was high grade PINs (Tables 1, 2). Desmoplastic reaction, characterized by the presence of larger stromal cells with increased formation of collagenous extracellular matrix, was detectable in prostate lesions of the *cPten* ^*-/-*^ *S* ^*+/+*^ and *cPten* ^*-/-*^S^+*/*-^ mice by 10 weeks and on, while it was practically absent in *cPten* ^*-/-*^S^-*/*-^ group until the 56-week time point. Analysis of normal *Ptenfloxed* */* *Survivinfloxed* (*Pten* ^*f/f*^ *S* ^*f/f*^) control tissue sections at various time points consistently showed the presence of regular prostatic glandular structures (Table S3).

**Table 1:** Prostate pathology of *cPten* ^*-/-*^ mice with monoallelic or biallelic deletion of *survivin*.

**Genotype**	**Age group (weeks)**	**Total no. of animals**	**No. of animals with PIN**	**No. of animals with early Ca to AdCa**
*cPten* ^*-/-*^	10 (9-11)	4	4 (1 LG, 3 HG)	1
	21 (19-23)	6	6 (HG)	1
	37 (34-41)	6	6 (HG)	3
	56 (51-62)	4	4 (HG)	4
*cPten* ^*-/-*^ *S* ^*+/-*^	10 (9-11)	4	4 (3 LG, 1 HG)	0
	21 (19-23)	5	5 (HG)	2
	37 (34-41)	5	5 (HG)	4
	56 (51-62)	5	5 (HG)	4
*cPten* ^*-/-*^ *S* ^*-/-*^	10 (9-11)	5	5 (LG)	0
	21 (19-23)	5	5 (HG)	0
	37 (34-41)	5	5 (HG)	0
	56 (51-62)	5	5 (HG)	0

Abbreviations: PIN, prostatic intraepithelial neoplasm PIN 1 and PIN2, low grade (LG) PINs; PIN3 and PIN4, high grade (HG) PINs; Early cancer, microscopic cancer; Adca, adenocarcinoma. Lesion classifications were as described [28-30]

**Table 2:** Details of prostate pathology observed in the *cPten* ^*-/-*^ *S* ^*-/-*^group.

**Animal No.**	**Age (wks)**	**Pathology**			**Remarks**
		**AP**	**VP**	**DLP**	
5496	9.4	PIN1	PIN1	PIN1	Few atypical cells (AP), single atypical (hypertrophic, polyploid)
					cells, some exfoliated (VP, DLP)
5912	9.6	PIN1	PIN1	PIN1	Exfoliation, single atypical cells, focal hyperplasia (AP), single
					atypical cells, exfoliation (VP), single atypical cells, hypertrophy,
					exfoliation (DLP)
5927	10	PIN1	PIN1	PIN2	Mainly RS, few PIN1, single atypical cell (AP), single atypical
					cells (VP), small PIN2 with large atypical cells, no desmoplastic
					stroma, exfoliation (DLP)
5928	10	RS	PIN1	PIN2	Areas of exfoliation, single atypical cells (AP), few atypical cells,
					exfoliation (DLP)
5812	11	RS	PIN1	PIN2	Some exfoliation (AP), few atypical/apoptotic/exfoliated cells
					(VP), small areas of enlarged (nucleus and cytoplasm) cells,
					some exfoliation, rare PIN2 (DLP)
5498	18.6	PIN1	PIN1	PIN3	Few atypical cells, exfoliation (AP), few large atypical cells,
					hypertrophy, exfoliation (VP), PINs with large atypical and
					apoptotic cells (DLP)
5753	19.7	PIN2	PIN1	PIN3	Small PIN2 (AP), large vacuolized cells filling up the ducts-
					hypertrophy (AP, DLP), few atypical cells (AP, VP)
5754	19.7	PIN1	PIN1	PIN3	Modest hyperplasia, some exfoliation (AP), few atypical cells, as
					in other cases such cells can be hyperploid (VP), large atypical
					cells, some of them are exfoliated, moderate hyperlasia (DLP)
5757	19.7	PIN3	PIN2	PIN3	Few large atypical cells (AP, VP), hyperplasia/hypertrophy (AP,
					VP, DLP), some apoptosis (DLP)
5774	19.7	PIN1	PIN1	PIN3	Single atypical cells, exfoliation (AP), large atypical cells but no
					no stratification/piling up (VP), hyperplasia, PIN3, single atypical
					cells, no desmoplastic reaction (DLP)
5675	39.3	PIN1	PIN2	PIN3	Single atypical cells (AP, VP), areas of hypertrophy (VP),
					hypertrophy/hyperplasia, some apoptosis, no desmoplasia (DLP)
5678	39.3	PIN1	PIN2	PIN3	Hypertrophy (AP), few large atypical cells (VP), hyperplasia/
					hypertrophy (VP, DLP), some apoptosis (DLP)
5679	39.3	PIN1	PIN1	PIN3	Few atypical cells (AP), single atypical and hypertrophic cells
					(VP), hypertrophy/hyperplasia, some apoptosis (DLP)
5683	39.3	PIN3	PIN4	PIN3	Few large atypical cells/hypertrophy (AP, VP), hyperplasia (VP),
					apoptosis (VP, DLP)
5681	39.4	PIN3	PIN4	PIN3	Massive exfoliation (AP)
5628	54	PIN3	PIN3	PIN4	Hypertrophy/hyperplasia, diffusely located atypical cells (VP)
5491	54.6	PIN3	PIN4	PIN3	Areas of hyperplasia (AP), apoptosis (VP)
5584	54.6	PIN2	PIN4	PIN4	Hypertrophy/hyperplasia, few atypical cells (AP)
5580	54.9	PIN3	PIN4	PIN4	Intraglandular focal necrosis (DLP)
5581	54.9		PIN4	PIN4	

Abbreviations: RS, regular structures as seen in the normal mouse prostate; PIN, prostatic intraepithelial neoplasm PIN 1 and PIN2, low grade (LG) PINs; PIN3 and PIN4, high grade (HG) PINs; Early cancer, microscopic cancer; Adca, adenocarcinoma. Lesion classifications were as described (28-30)

### Characteristics of expression of cellular markers in the prostate tumors formed in the conditional Pten deletion mice with heterozygous or homozygous deletion of Survivin gene

**Figure 4:**
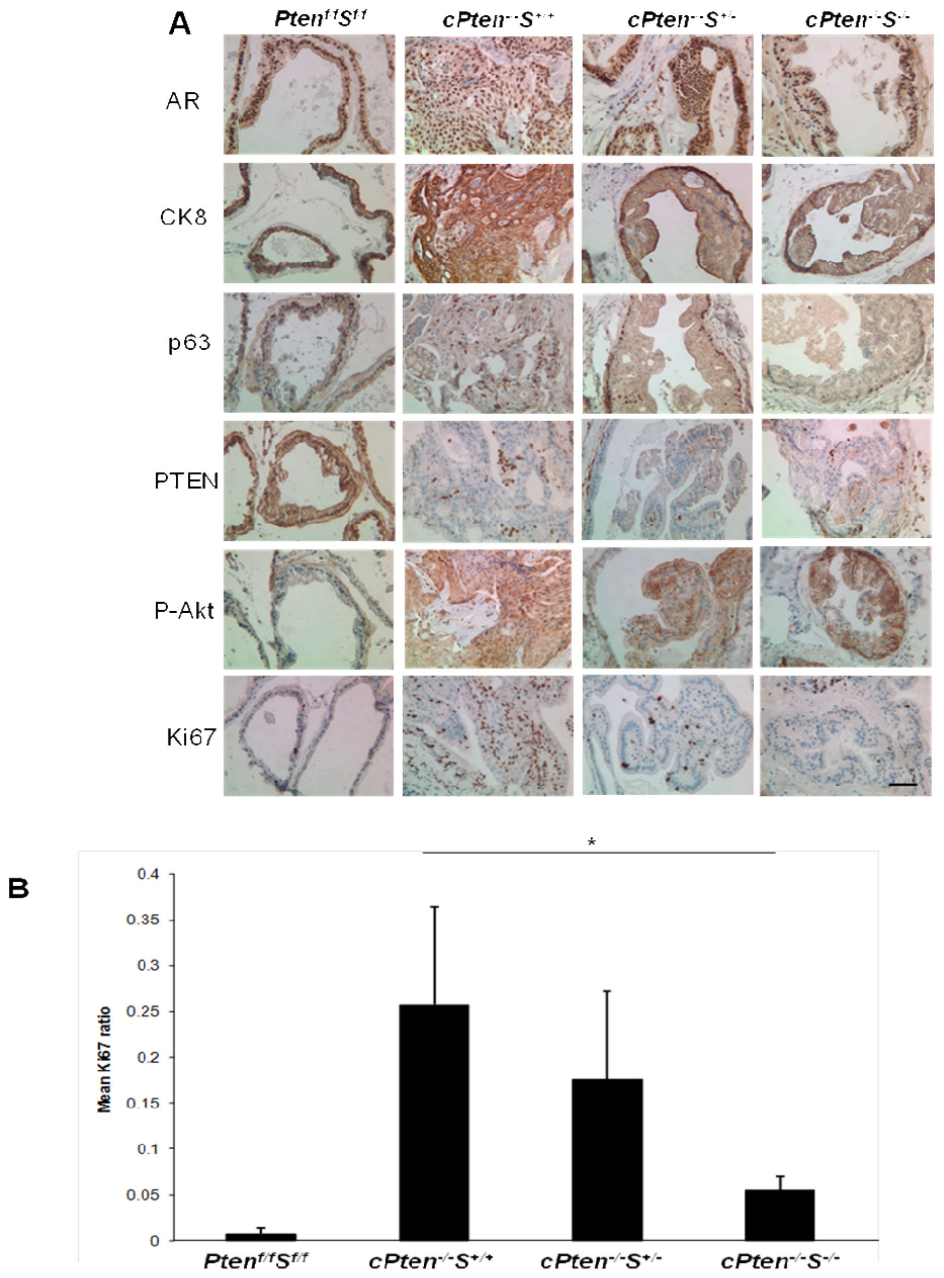
Representative pattern of expression of cellular markers in prostates of conditional *Pten* knockout mice with heterozygous and homozygous deletion of *Survivin*. (A) Immunostaining of dorsolateral prostate lobes of animals from the 56-week old group of different genotypes using antibodies against androgen receptor (AR), cytokeratin 8 (CK8), basal epithelial marker p63, PTEN, phosphorylated Akt (P-Akt), and Ki67. All images were taken at 400x magnification. Bar, 50 µm. (B) Comparison of proliferation index as assessed by Ki67 staining of dorsolateral lobes of the 56-week old group. * P < 0.05.

**Figure 5:**
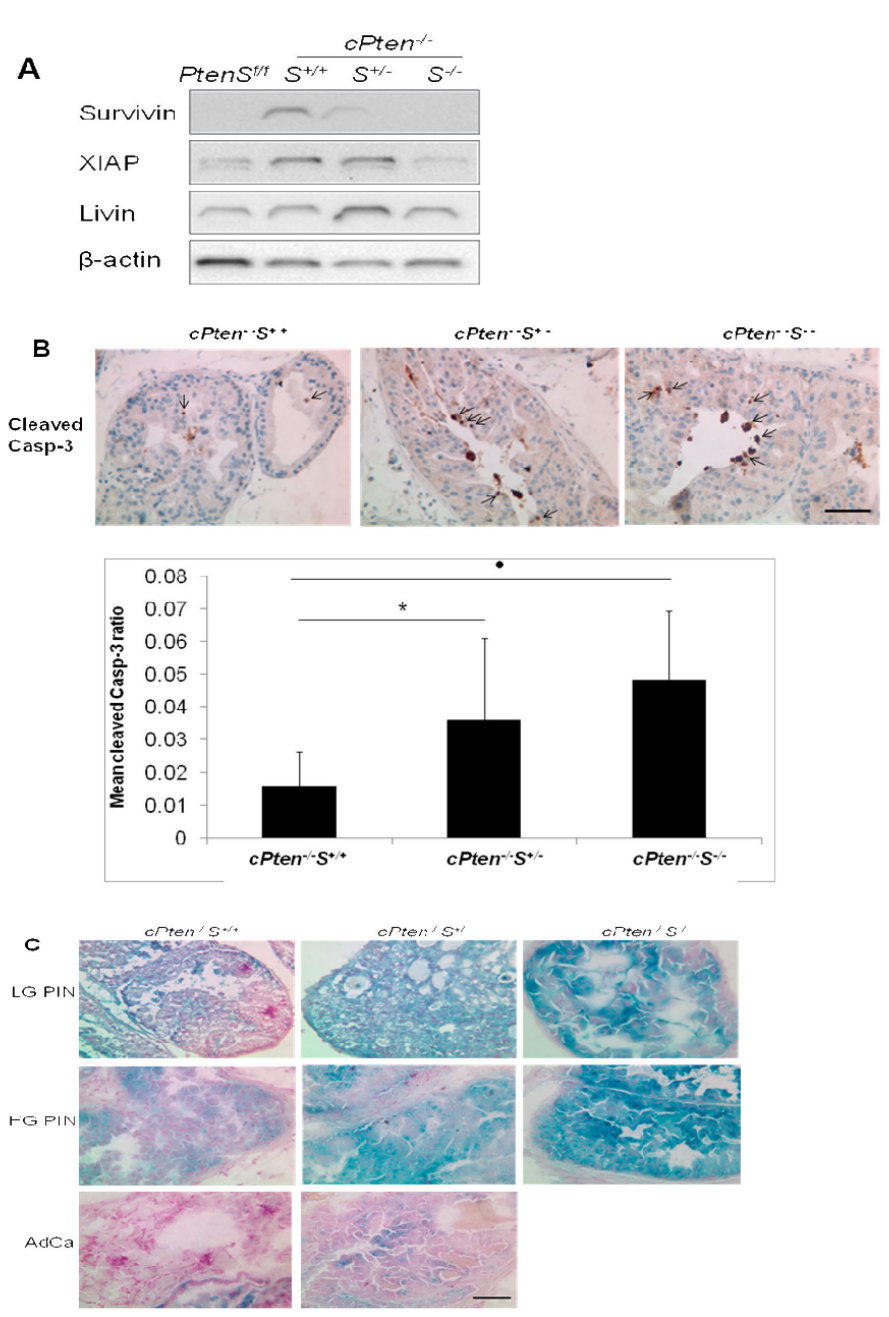
Effects of *Survivin* deletion on other molecular and cellular parameters. (A) Representative Western blot analysis of dorsolateral prostates from the 37-week old group showed that XIAP level was down-regulated in prostate tissues lacking both alleles of *Survivin*, while Livin was relatively unaffected. A similar pattern of XIAP expression was also observed when samples were obtained from in 10 and 21 week- old groups. (B) Detection of higher levels of cleaved-caspase 3 expression, indicated by arrows, in the PIN lesions from the conditional *Pten* deleted prostate tumors lacking single or both alleles of *Survivin* compared to tumors with intact *Survivin*. * P < 0.05; · P <0.01. (C) Illustration of senescence-activated β-galactosidase staining results. An increase in senescence was indicated in the conditional *Pten* deleted prostate samples with complete inactivation of *Survivin* especially at the high-grade PIN stage compared to samples with intact and monoallelic deletion of *Survivin*. Note only low level of senescence in prostate adenocarcinoma tissues of conditional *Pten* knockout mice with either with intact and single deletion of *Survivin*. PINs are denoted as low-grade (LG) or high-grade (HG), and adenocarcinoma as AdCa. All images were taken at 400x magnification. Bar, 50 µm.

**Figure 6:**
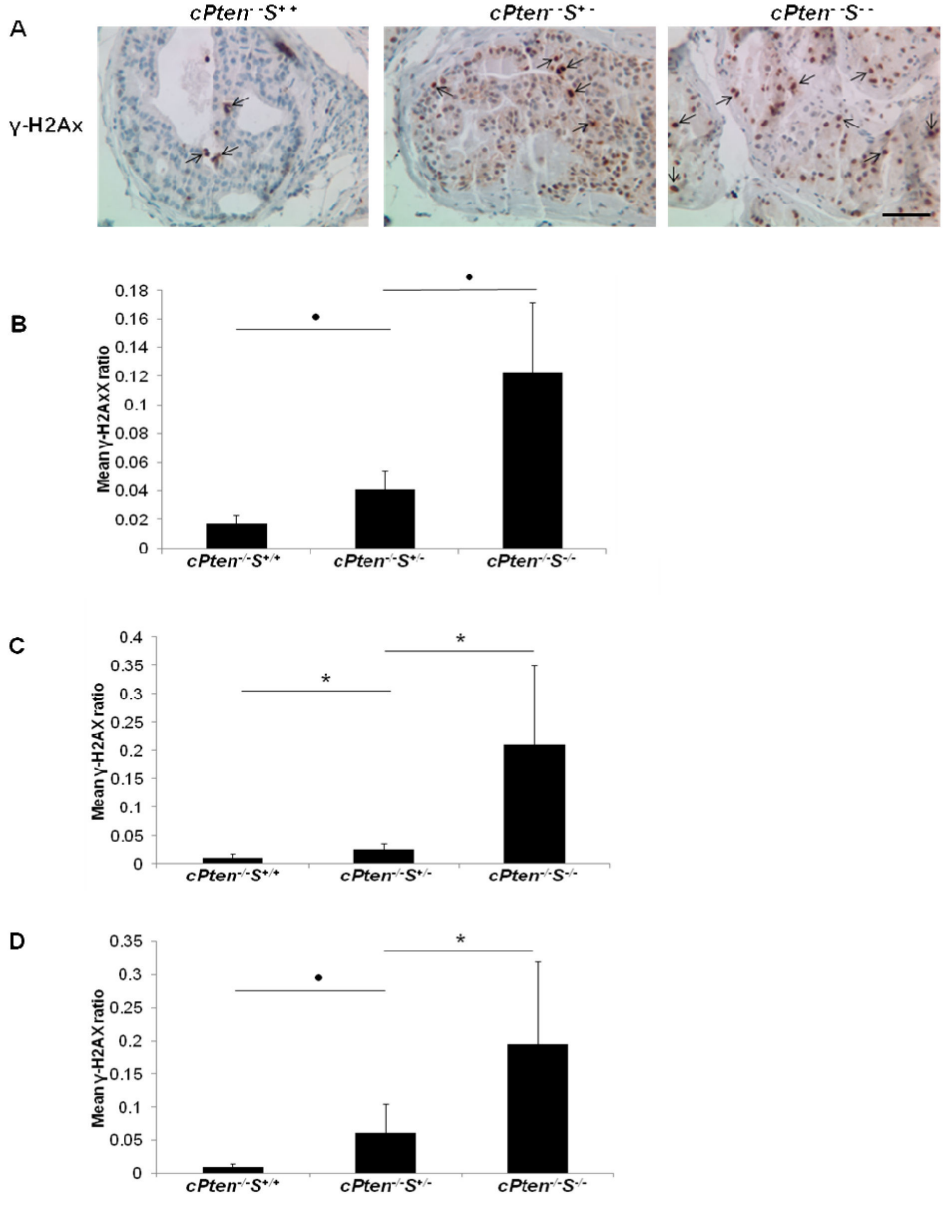
Assessment of γ-H2AX immunostaining in prostate tissue sections. (A) Representative view of γ-H2AX expression, indicated by arrows, in the prostate samples at 10 weeks from the conditional *Pten* knockout mice with single or double deletion of *Survivin* as compared to corresponding samples with intact *Survivin*. Images were taken at 400x magnification. Bar, 50 µm. (B) (C), and (D) show the results of quantitation of γ-H2AX positive cells in samples at 10, 21 and 37 weeks, respectively. * P < 0.05; · P <0.01.

Immunohistochemistry results (Figure 4A) showed that the prostate cells making up glandular structures in all four groups of mice stained positive for androgen receptor (AR), luminal epithelial marker cytokeratin 8 (CK8), and basal epithelial cell marker p63. Knock-down of *Pten* specifically in the prostate epithelium was confirmed by the distinctive lack of PTEN protein staining in that area compared to the abundance of PTEN expression in the surrounding stroma. Correspondingly, the level of detection of phosphorylated AKT was elevated in all mouse prostate tissue sections with conditional *Pten* deletion, regardless of the status of survivin expression. A significant downregulation in the expression of Ki67 was observed in prostate tissues of *cPten* ^*-/-*^S^-*/*-^ compared to *cPten* ^*-/-*^ *S* ^*+/+*^ or *cPten* ^*-/-*^ *S* ^*+/-*^. Representative results illustrating these observations are shown in Figure 4A, B. The results of Ki67 staining suggested a role of survivin in cell proliferation. This observation was also consistent with the finding that the tumor size in *cPten* ^*-/-*^S^-*/*-^ being strikingly smaller in comparison to those of either *cPten* ^*-/-*^ *S* ^*+/+*^ and *cPten* ^*-/-*^ *S* ^*+/-*^ (Figure 3A–D). Considering that an increased occurrence of apoptotic cells was noted in the histological analyses of samples from *cPten* ^*-/-*^S^-*/*-^ group (Table 2), we undertook a further assessment by immunohistochemistry using an antibody against cleaved caspase-3. We found that the activated caspase-3 expression level was indeed higher in the prostates of cPten^-/-^S^-/-^ compared to those of the other groups (Figure 5B). This is illustrated with PIN2 lesions where the increase was most pronounced. Another noteworthy histologic observation was that the prostatic lesions in the *cPten* ^*-/-*^S^-*/*-^ mice frequently contained, in addition of apoptotic cells, other enlarged, atypical cells indicative of increased incidence of cellular senescence (Table 2, as compared to Supplemental Tables 1-3). This assumption was then tested by an *in situ* assay of senescence-associated β-galactosidase enzyme activity on the frozen tissue sections, as described [26]. It appeared that a higher proportion of cells exhibiting stronger staining (blue color) for β-galactosidase was present in the *cPten* ^*-/-*^S^-*/*-^ tissues relative to either cPten^+/-^ or *cPten* ^*-/-*^ *S* ^*+/+*^ tissues, especially in high grade PIN stage (Figure 5C). Cells in the adenocarcinoma samples showed only minimal staining for senescence. A significant increase of phosphorylated H2AX, or γ-H2AX, was also detected by immunohistochemical analysis of the prostate samples of mice lacking a single or both alleles of *Survivin* compared to those with intact *Survivin* (Figure 6A). The extent of phosphorylation was positively correlated with the degree of *Survivin* deletion and was consistently observed throughout various time points (Figure 6B–D).

### Effects of *Survivin* knockout on the expression levels of other IAP members in the prostate tumor tissues

Western blot analysis revealed that XIAP, a member of the IAP family, displayed a similar expression level as survivin. Although, like survivin, its expression increased in the *Pten* deleted tumor tissues, XIAP appeared to be down-regulated with the loss of survivin expression. This type of corollary expression, however, was not observed with Livin, another IAP family member. Livin protein expression did increase in the lesions of *Pten*- deleted prostates, but there was no significant alteration in its expression in the cases when *Survivin* was deleted (Figure 5A).

## Discussion

In the cancer field, survivin stands as a unique member of the IAP family with essential roles in mitosis, cellular stress response and inhibition of cell death. It was, however, not known whether survivin plays a role in the development of the normal prostate and how this multi-functional protein might be relevant to its role in prostate carcinogenesis. For this purpose, we first determined if the organogenesis and growth of the prostate gland might be influenced by survivin. Mice with conditional inactivation of *Survivin* in prostate epithelium were generated by crossing mice of our *PB-Cre4* line [23] with floxed Survivin mice [22]. Through breeding and analyses of these mice at various ages up to one year, we demonstrate that homozygous inactivation of *Survivin* alleles in the epithelial cells of the prostate does not interfere with the development of fertile males harboring prostate gland with generally normal gross and microscopic anatomy. The role of survivin in prostate cancer genesis and progression was then investigated using the conditional biallelic *Pten* deletion model [17–19] by crossing the tumor model with the floxed Survivin allelic mice. Our contention was that prostate epithelium-specific Survivin nullizygous condition would likely enhance cellular apoptosis to counter prostate tumorigenesis. Here, using this combined model we provide evidence that loss of survivin inhibits progression of premalignant lesions to adenocarcinoma, and that the premalignant lesions exhibiting decreased proliferation index are composed of atypical cells, many of which exhibit increased hypertrophy and senescence.

Survivin expression was reported to be up-regulated in the early prostate tumor growth in both the conditional *Pten* knockout [16] and the TRAMP [31] mouse models. This implication of survivin in early pathogenesis is corroborated by our observation that deletion of either single or both alleles of *Survivin* can influence the phenotype of the PIN formed as early as 9 weeks of age. While all groups displayed low grade PIN formation, none of the four *cPten* ^*-/-*^S^-*/*-^ and only 1 of 4 *cPten* ^*-/-*^S^+*/*-^ mice was found to develop high grade PIN lesions at this age, compared to 3 out of 4 *cPten* ^*-/-*^S^+*/*+^ mice. In the age group of 56 weeks, 80-100% of the mice with intact or singly deleted *Survivin* alleles harbored adenocarcinoma, while none (0/5) of the mice with the homozygous *Survivin* deletion developed adenocarcinoma, although high grade PINs were detected. In relation to the known influence of the genetic background and modifier genes on susceptibility to tumorigenesis, we believe that it is unlikely that the suppression of the progression to adenocarcinoma in our test model is due to variation in the mixed genetic background of the animals, because some of the *cPten* ^*-/-*^S^+*/*-^ mice developed adenocarcinoma at age as early as 21 weeks, whereas *cPten* ^*-/-*^S^-*/*-^ littermates with similar mixed background were free of cancer even at over one year age. Still, to increase the validity of the study, it will be necessary to breed all of the three pertinent strains, namely *PBCre-4*, *floxed Pten*, and the *floxed Survivin*, into a single genetic background that is also conducive for tumorigenesis. This major task, however, remains to be initiated. The question of whether the *cPten* ^*-/-*^S^-*/*-^ mice might manifest cancer on further aging was examined very recently. Of the three *cPten* ^*-/-*^S^-*/*-^ 72 week-old males examined subsequently, only one was found to display development of adenocarcinoma in the prostate (data not shown).

With respect to other histopathology parameters, hyperplasia was abundantly detected in the *cPten* ^*-/-*^S^-*/*-^ mice, but only rarely in *cPten* ^*-/-*^S^+*/*-^ or the control *cPten* ^*-/-*^S^+*/*+^ group. Furthermore, hypertrophy or enlargement of cells, and polyploidy or hyperploidy were relatively more frequent in the prostate samples from *cPten* ^*-/-*^S^-*/*-^ compared to the other groups. Survivin deficient cells have been reported to exhibit multiple nuclei *in vitro* and *in vivo*, consistent with the known role of survivin in the regulation of cytokinesis and cell division [11,22]. Another striking difference between single and biallelic deletion of *Survivin* was the lack of desmoplasia observed in the prostate tissue of *cPten* ^*-/-*^S^-*/*-^ mice younger than the 56 weeks age group that was prominently present in *cPten* ^*-/-*^S^+*/*+^ and *cPten* ^*-/-*^S^+*/*-^ samples from all four age groups tested. A reduction in the rate of cell proliferation was evident from the significant down-regulation of Ki67 expression in the specimens from the *cPten* ^*-/-*^S^-*/*-^ animals. This effect was the most striking with the 10 weeks age group, whether the samples were from the monoallelic or biallelic Survivin knockout animals. However, with time, the degree of proliferation became somewhat parallel with the extent of *Survivin* insufficiency.

Another noteworthy point is that we found that the degree of *Survivin* deletion in the tumors differentially affected the detectable levels of expression of some other IAP family members. For example, the expression of XIAP, but not Livin was reduced. This phenomenon could probably be attributed to the function of the complex known to form between survivin and XIAP that stabilizes XIAP and protects it from ubiquitin-dependent degradation [3]. The survivin-XIAP complex was also described to enhance XIAP’s capacity to inhibit caspases and to facilitate tumor growth *in vivo* [3,32]. The reduction of XIAP in the setting of *Survivin* deficiency may therefore further protect against tumorigenesis.

Consistent with previous findings in various other study systems [33–35], we observed an increase in apoptosis as assessed by the expression level of activated caspase-3 in the prostatic lesions of the conditional *Pten* knockout mice lacking both alleles of *Survivin*. This effect was most pronounced and significant at the low grade PIN stage. We also detected a correlation between loss of survivin expression and senescence in *Pten*-deleted prostate tumor tissues. By using the simplified method of staining for senescence-associated β-galactosidase in the tissue sections [36], we attempted to measure the extent of senescence induction in the prostate of the various groups of mice. Presence of senescent cells was readily detected in conditional *Pten* deleted mice with intact *Survivin* at low grade and high grade PIN stages but minimally when the tumor had advanced to adenocarcinoma. This finding is consistent with previous reports that senescence may be an initial barrier in cancer development [37–39], and that senescent cells exist in premalignant tumors but not in malignant ones [39]. The pattern of senescence observed in the group with monoallelic and biallelic Survivin loss was not detectably different from the control tumor group at the low grade PIN stage. However, at the high grade PIN stage, lesions of the group with single and especially double deletion of *Survivin* loss exhibited a higher proportion of senescent cells and greater intensity of staining. Since γ-H2AX, whose expression is induced in cells following initial DNA fragmentation, is considered as a cellular senescence marker [40–42] our data on β-galactosidase criterion appears to be supported by the results of γ-H2AX staining. Clearly, the increase in the detection of γ-H2AX-positive cells is found to be associated with the degree of *Survivin* deletion in the conditional *Pten* knockout mice. However, it remains unknown to what extent this increased staining may be subscribed to senescence vs. DNA double strand breaks and induced γ-H2AX that occurs during apoptosis [43]. Although an association of survivin loss with senescence in the lesions is indicated, not clear is whether this relationship is direct or secondary to the loss of the multi-functionality of the survivin protein that may be critical for the progression of the preneoplastic lesions to cancer. It is also possible that the defects in microtubule assembly, loss of mitotic spindles, and formation of multinucleated cells, the abnormalities that are triggered by the loss of survivin [2,9,11] could make the cells prone for senescence. Further studies into the effect of survivin on cellular senescence would be important.

In summary, the results of our investigation on the role of survivin in prostate cancer progression using a double conditional *Pten* and *Survivin* mouse model is particularly significant because it led to insights of the direct impact of *Survivin* deletion occurring simultaneously with that of *Pten* deletion in the process of tumorigenesis. Evidence is obtained to link a supporting role of survivin in the progression of PIN lesions to adenocarcinoma of the prostate in the model system. Additionally, it is apparent that lesions formed in the absence of survivin are variant in microscopic phenotypes with hallmarks of hypertrophy, exfoliation, apoptosis and senescence. These findings offer a potential for future pre-clinical or clinical investigation for the control of PIN lesions. It is projected from the findings of this study that inhibition of survivin activity may retard or block progression of the PIN lesions, and, thereby extending the therapeutic window for prostate cancer.

## Supporting Information

Figure S1 Breeding scheme for prostate-specific Survivin deleted mice. (A) Conditional deletion of Survivin in murine prostate. (B) Double conditional knockout of Pten and Survivin in murine prostate. The genotypes of the mice outlined with the red boxes were used for the study.
(PPTX)

Table S1 Prostate pathology observed in the *cPten* ^*-/-*^ *S* ^*+/+*^ control tumor group. Abbreviations: AP, VP, DLP, anterior, ventral and dorsolateral prostates, respectively; PIN, prostatic intraepithelial neoplasms; PIN1 and PIN2, low grade PINs; PIN3 and PIN4, high grade PINs; Early cancer, microscopic cancer, AdCa, adenocarcinoma.
(PPTX)

Table S2 Prostate pathology observed in the *cPten* ^*-/-*^ *S* ^*+/-*^ experimental group. Abbreviations: AP, VP, DLP, anterior, ventral and dorsolateral prostates, respectively; PIN, prostatic intraepithelial neoplasms; PIN1 and PIN2, low grade PINs; PIN3 and PIN4, high grade PINs; Early cancer, microscopic cancer, AdCa, adenocarcinoma.
(PPTX)

Table S3 Description of histologic evaluation of the prostate in the *Pten* ^*f/f*^ *S* ^f/f^ control group. Abbreviations: AP, VP, DLP, anterior, ventral and dorsolateral prostates, respectively; RS, regular structures as seen in the normal mouse prostate.
(PPTX)
