# Supplementary material for: Loss of Survivin in the Prostate Epithelium Impedes Carcinogenesis in a Mouse Model of Prostate Adenocarcinoma
Source: PLoS One. 2013 Jul 31;8(7):e69484. doi: 10.1371/journal.pone.0069484 (PMC3729965; doi:10.1371/journal.pone.0069484)
Supplement: Figure S1 — (A) Conditional deletion of Survivin in murine prostate. (B) Double conditional knockout of Pten and Survivin in murine prostate. The genotypes of the mice outlined with the red boxes were used for the study. (PPTX) [file pone.0069484.s001.pptx]

## Slide 1
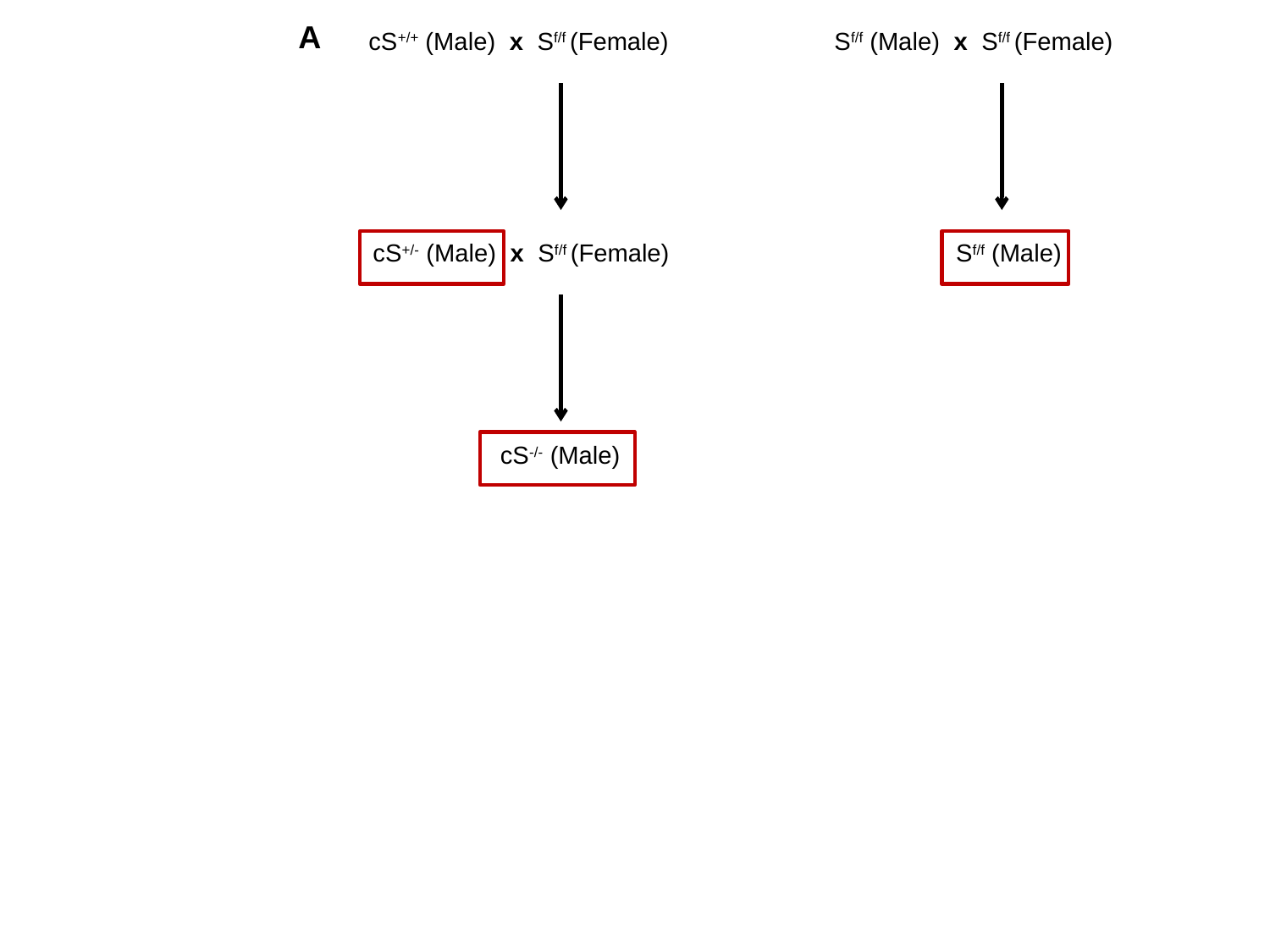

A
 cS+/+ (Male) x Sf/f (Female)
 Sf/f (Male) x Sf/f (Female)
 cS+/- (Male) x Sf/f (Female)
 Sf/f (Male)
 cS-/- (Male)

## Slide 2
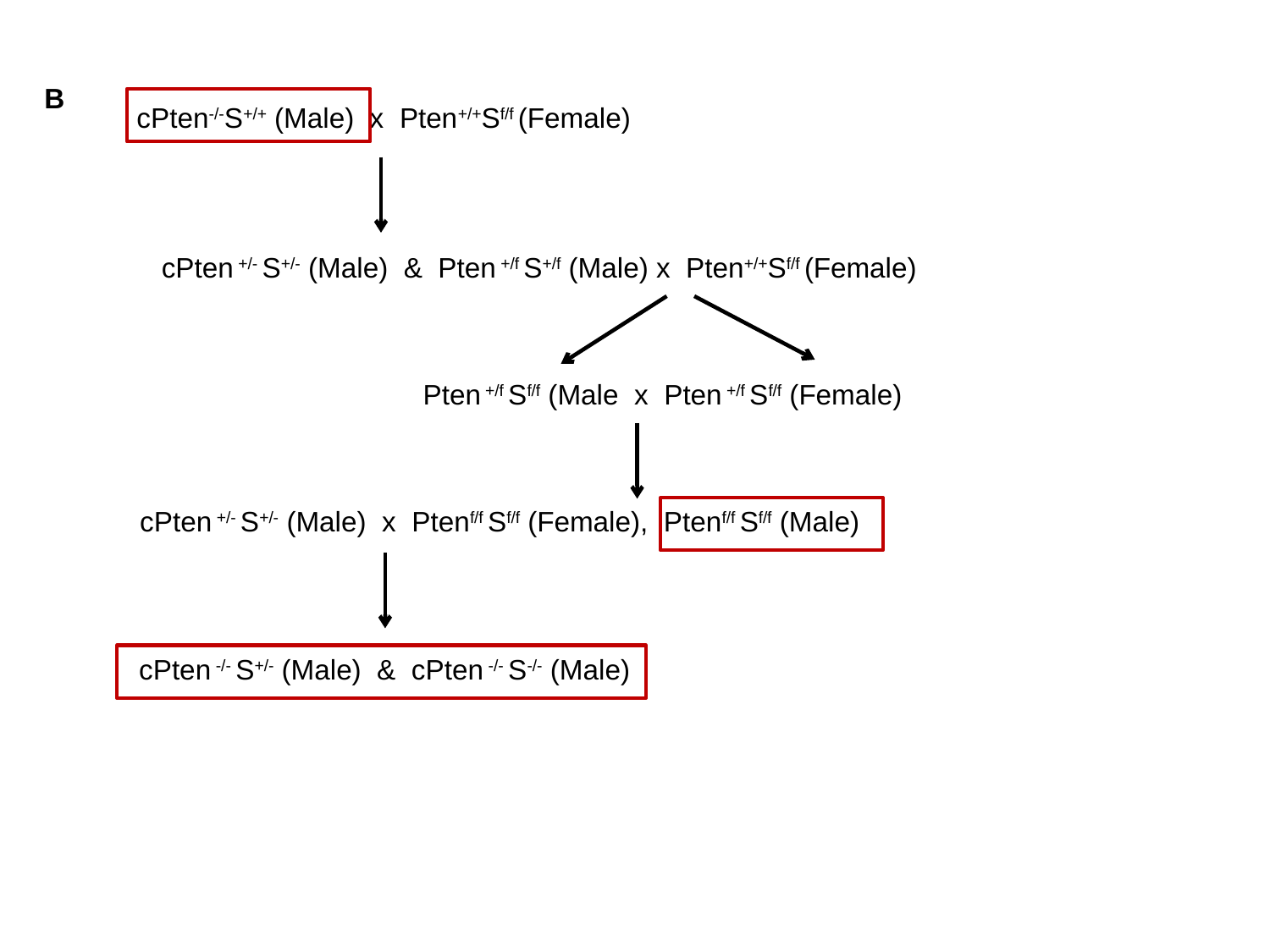

B
 cPten-/-S+/+ (Male) x Pten+/+Sf/f (Female)
 cPten +/- S+/- (Male) & Pten +/f S+/f (Male) x Pten+/+Sf/f (Female)
Pten +/f Sf/f (Male x Pten +/f Sf/f (Female)
cPten +/- S+/- (Male) x Ptenf/f Sf/f (Female), Ptenf/f Sf/f (Male)
cPten -/- S+/- (Male) & cPten -/- S-/- (Male)
