# Supplementary material for: Loss of Survivin in the Prostate Epithelium Impedes Carcinogenesis in a Mouse Model of Prostate Adenocarcinoma
Source: PLoS One. 2013 Jul 31;8(7):e69484. doi: 10.1371/journal.pone.0069484 (PMC3729965; doi:10.1371/journal.pone.0069484)
Supplement: Table S1 — Abbreviations: AP, VP, DLP, anterior, ventral and dorsolateral prostates, respectively; PIN, prostatic intraepithelial neoplasms; PIN1 and PIN2, low grade PINs; PIN3 and PIN4, high grade PINs; Early cancer, microscopic cancer, AdCa, adenocarcinoma. (PPTX) [file pone.0069484.s002.pptx]

## Slide 1
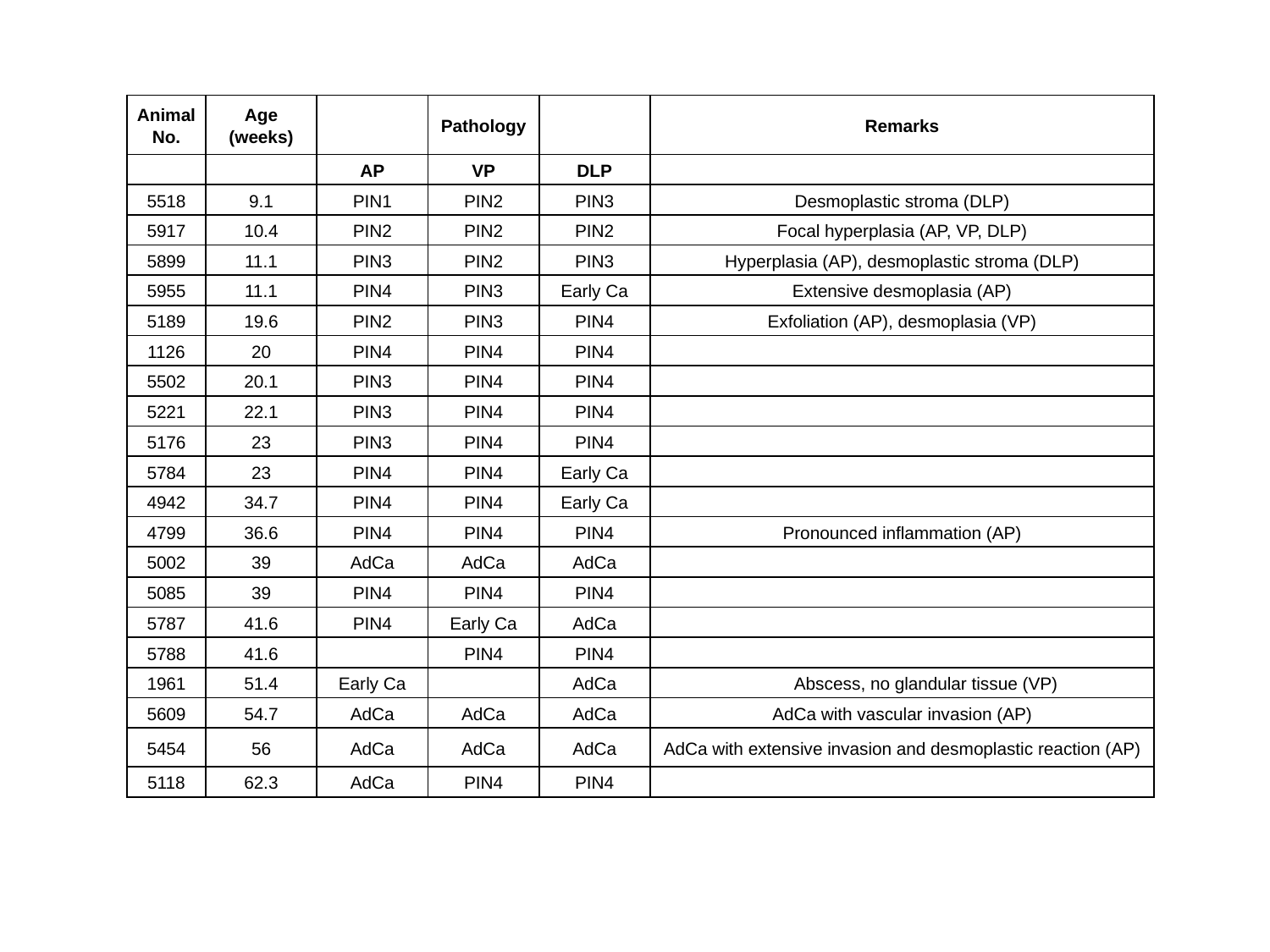

| Animal No. | Age (weeks) | | Pathology | | Remarks |
| --- | --- | --- | --- | --- | --- |
| | | AP | VP | DLP | |
| 5518 | 9.1 | PIN1 | PIN2 | PIN3 | Desmoplastic stroma (DLP) |
| 5917 | 10.4 | PIN2 | PIN2 | PIN2 | Focal hyperplasia (AP, VP, DLP) |
| 5899 | 11.1 | PIN3 | PIN2 | PIN3 | Hyperplasia (AP), desmoplastic stroma (DLP) |
| 5955 | 11.1 | PIN4 | PIN3 | Early Ca | Extensive desmoplasia (AP) |
| 5189 | 19.6 | PIN2 | PIN3 | PIN4 | Exfoliation (AP), desmoplasia (VP) |
| 1126 | 20 | PIN4 | PIN4 | PIN4 | |
| 5502 | 20.1 | PIN3 | PIN4 | PIN4 | |
| 5221 | 22.1 | PIN3 | PIN4 | PIN4 | |
| 5176 | 23 | PIN3 | PIN4 | PIN4 | |
| 5784 | 23 | PIN4 | PIN4 | Early Ca | |
| 4942 | 34.7 | PIN4 | PIN4 | Early Ca | |
| 4799 | 36.6 | PIN4 | PIN4 | PIN4 | Pronounced inflammation (AP) |
| 5002 | 39 | AdCa | AdCa | AdCa | |
| 5085 | 39 | PIN4 | PIN4 | PIN4 | |
| 5787 | 41.6 | PIN4 | Early Ca | AdCa | |
| 5788 | 41.6 | | PIN4 | PIN4 | |
| 1961 | 51.4 | Early Ca | | AdCa | Abscess, no glandular tissue (VP) |
| 5609 | 54.7 | AdCa | AdCa | AdCa | AdCa with vascular invasion (AP) |
| 5454 | 56 | AdCa | AdCa | AdCa | AdCa with extensive invasion and desmoplastic reaction (AP) |
| 5118 | 62.3 | AdCa | PIN4 | PIN4 | |
