# Supplementary material for: Loss of Survivin in the Prostate Epithelium Impedes Carcinogenesis in a Mouse Model of Prostate Adenocarcinoma
Source: PLoS One. 2013 Jul 31;8(7):e69484. doi: 10.1371/journal.pone.0069484 (PMC3729965; doi:10.1371/journal.pone.0069484)
Supplement: Table S2 — Abbreviations: AP, VP, DLP, anterior, ventral and dorsolateral prostates, respectively; PIN, prostatic intraepithelial neoplasms; PIN1 and PIN2, low grade PINs; PIN3 and PIN4, high grade PINs; Early cancer, microscopic cancer, AdCa, adenocarcinoma. (PPTX) [file pone.0069484.s003.pptx]

## Slide 1
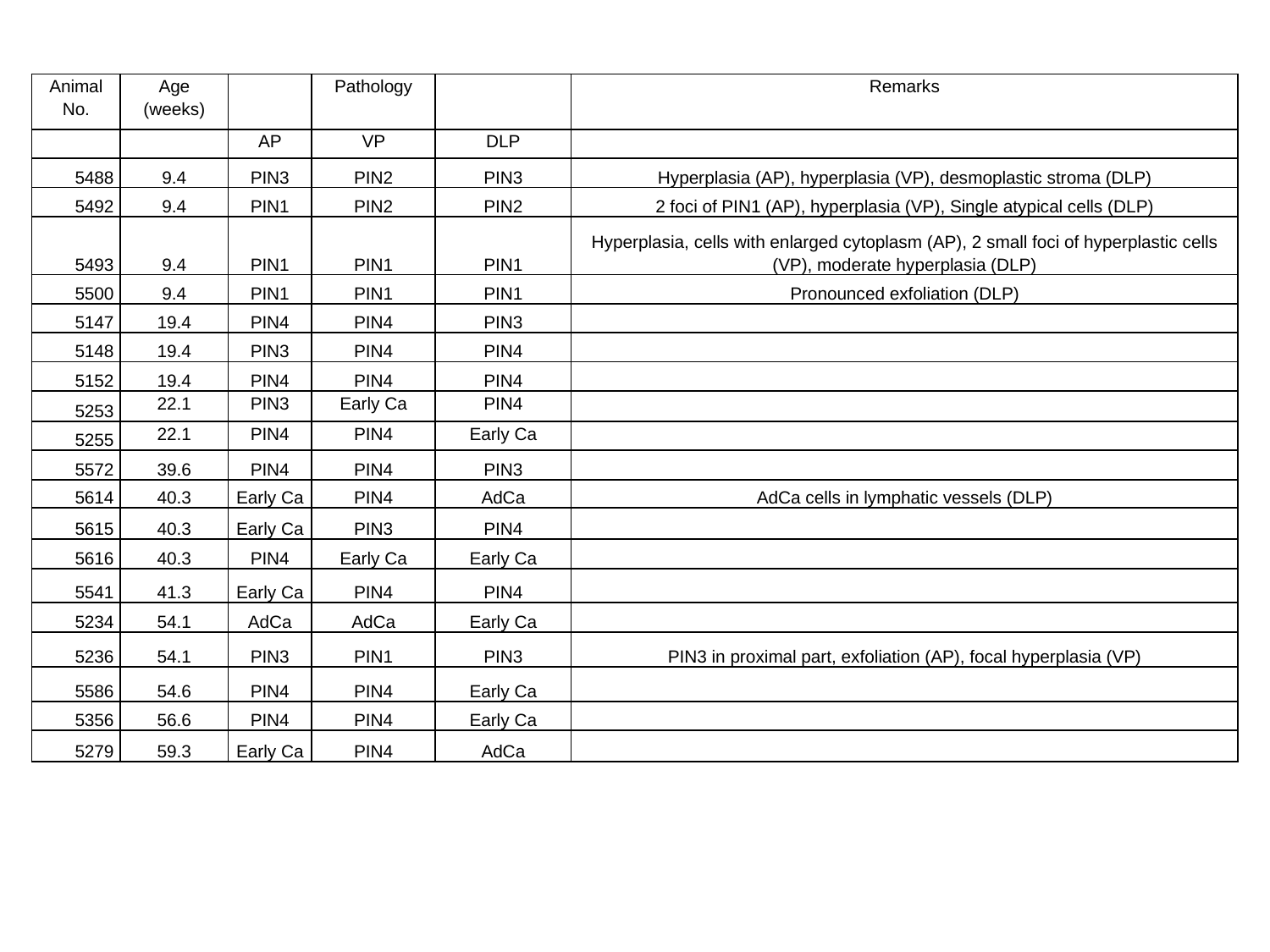

| Animal No. | Age (weeks) | | Pathology | | Remarks |
| --- | --- | --- | --- | --- | --- |
| | | AP | VP | DLP | |
| 5488 | 9.4 | PIN3 | PIN2 | PIN3 | Hyperplasia (AP), hyperplasia (VP), desmoplastic stroma (DLP) |
| 5492 | 9.4 | PIN1 | PIN2 | PIN2 | 2 foci of PIN1 (AP), hyperplasia (VP), Single atypical cells (DLP) |
| 5493 | 9.4 | PIN1 | PIN1 | PIN1 | Hyperplasia, cells with enlarged cytoplasm (AP), 2 small foci of hyperplastic cells (VP), moderate hyperplasia (DLP) |
| 5500 | 9.4 | PIN1 | PIN1 | PIN1 | Pronounced exfoliation (DLP) |
| 5147 | 19.4 | PIN4 | PIN4 | PIN3 | |
| 5148 | 19.4 | PIN3 | PIN4 | PIN4 | |
| 5152 | 19.4 | PIN4 | PIN4 | PIN4 | |
| 5253 | 22.1 | PIN3 | Early Ca | PIN4 | |
| 5255 | 22.1 | PIN4 | PIN4 | Early Ca | |
| 5572 | 39.6 | PIN4 | PIN4 | PIN3 | |
| 5614 | 40.3 | Early Ca | PIN4 | AdCa | AdCa cells in lymphatic vessels (DLP) |
| 5615 | 40.3 | Early Ca | PIN3 | PIN4 | |
| 5616 | 40.3 | PIN4 | Early Ca | Early Ca | |
| 5541 | 41.3 | Early Ca | PIN4 | PIN4 | |
| 5234 | 54.1 | AdCa | AdCa | Early Ca | |
| 5236 | 54.1 | PIN3 | PIN1 | PIN3 | PIN3 in proximal part, exfoliation (AP), focal hyperplasia (VP) |
| 5586 | 54.6 | PIN4 | PIN4 | Early Ca | |
| 5356 | 56.6 | PIN4 | PIN4 | Early Ca | |
| 5279 | 59.3 | Early Ca | PIN4 | AdCa | |
