# Supplementary material for: Loss of Survivin in the Prostate Epithelium Impedes Carcinogenesis in a Mouse Model of Prostate Adenocarcinoma
Source: PLoS One. 2013 Jul 31;8(7):e69484. doi: 10.1371/journal.pone.0069484 (PMC3729965; doi:10.1371/journal.pone.0069484)
Supplement: Table S3 — Abbreviations: AP, VP, DLP, anterior, ventral and dorsolateral prostates, respectively; RS, regular structures as seen in the normal mouse prostate. (PPTX) [file pone.0069484.s004.pptx]

## Slide 1
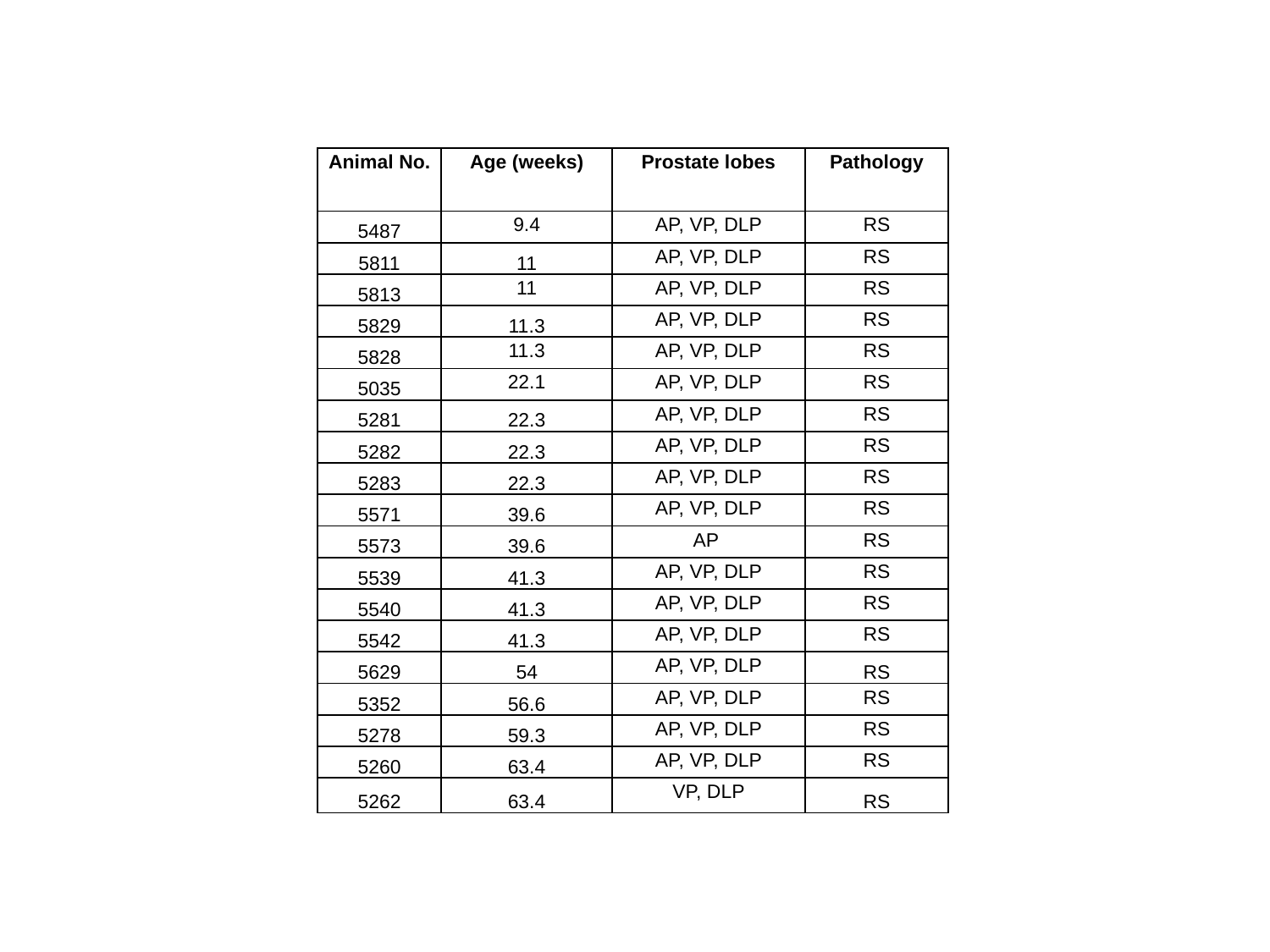

| Animal No. | Age (weeks) | Prostate lobes | Pathology |
| --- | --- | --- | --- |
| 5487 | 9.4 | AP, VP, DLP | RS |
| 5811 | 11 | AP, VP, DLP | RS |
| 5813 | 11 | AP, VP, DLP | RS |
| 5829 | 11.3 | AP, VP, DLP | RS |
| 5828 | 11.3 | AP, VP, DLP | RS |
| 5035 | 22.1 | AP, VP, DLP | RS |
| 5281 | 22.3 | AP, VP, DLP | RS |
| 5282 | 22.3 | AP, VP, DLP | RS |
| 5283 | 22.3 | AP, VP, DLP | RS |
| 5571 | 39.6 | AP, VP, DLP | RS |
| 5573 | 39.6 | AP | RS |
| 5539 | 41.3 | AP, VP, DLP | RS |
| 5540 | 41.3 | AP, VP, DLP | RS |
| 5542 | 41.3 | AP, VP, DLP | RS |
| 5629 | 54 | AP, VP, DLP | RS |
| 5352 | 56.6 | AP, VP, DLP | RS |
| 5278 | 59.3 | AP, VP, DLP | RS |
| 5260 | 63.4 | AP, VP, DLP | RS |
| 5262 | 63.4 | VP, DLP | RS |
